# Can Transgression Define Identity in Educational Settings? A Basque-Based Framework for Identity-in-Interaction

**DOI:** 10.3389/fpsyg.2019.01741

**Published:** 2019-07-30

**Authors:** Elizabeth Pérez-Izaguirre

**Affiliations:** Department of Didactics and School Organization, University of the Basque Country (UPV/EHU), Bilbao, Spain

**Keywords:** interaction, identity, transgression, multi-ethnic, Basque, education, ethnography

## Abstract

Interaction in educational environments might refer to a set of relationships between individuals in a school system. These links can be considered within a power-relations framework that includes the role of each of the subjects in a school and its community. This paper focuses on “transgressive” interactions involving adolescent students from diverse ethnic backgrounds in a Basque secondary school and relies on the concept of identity-in-interaction from a sociocultural approach, according to which, identity is constituted in a process of exchange between two or more parties. This research is drawn from the results of an ethnographic study conducted between July 2015 and June 2016 in a Basque secondary school attended by a high proportion of immigrant students, which shares characteristics with the broader Basque educational context. The methods used to collect data included documentary analysis, 9-months of participant observation, 36 in-depth interviews and four focus groups. Transgression in this context refers to the act of questioning socially established limits of behavior, which is considered typical during adolescent years. I categorize three types of student interaction as transgressive: personal, civic, and social limit transgressions, which involve challenges to peer-interpersonal, institutional, and community rules of interaction, respectively. In the Basque Country there are two official languages, Basque and Spanish, and students are instructed in both languages. They must daily face the particularities of a bilingual society where Basque is still a minority language. Community transgression is noteworthy, as immigrant students resisted the rule enforcing Basque instruction, leading to intercultural conflict with teachers. The study argues that identity can be constructed through transgression: immigrants’ refusing to learn Basque is a matter of rebellion that acts as an identity marker. This study contributes to the discussion of identity-in-interaction. Based on empirical data it uses the framework of transgression in multi-ethnic educational environments to consider community languages in a broader power-relations framework.

## Introduction

This paper is part of a larger ethnographic study researching multi-ethnic student-to-student and teacher-to-student interactions in Basque secondary education. The Basque Country is located in the south of France and north of Spain and the school I refer to is located in the Basque Autonomous Community (BAC), one of the regions of the Basque Country in Spain where both Basque and Spanish have official status. Today, institutions in the BAC reinforce Basque language, but during Franco’s dictatorship between the late 1930s and mid 1970s speaking Basque was prohibited (Cenoz, 2009). Basque remains a minority language across most areas in the Basque Country and in the BAC all public and most private schools promote its instruction (Echeverria, 2003; Martínez, 2014). Many school and social rituals are performed in Basque to strengthen links with the Basque community and offer cultural reparation for the period when it was prohibited. Consequently, it is an important identity marker for a great part of the Basque community, who often feel it is their duty to protect and maintain it (Urla, 2012). Basque is also a source of social and linguistic conflict, as some people do not support activities in Basque or do not feel their identity relates to it.

Today, this complex bilingual social and educational context faces another challenge, as the BAC has received a considerable immigrant population in recent years^1^. In the Basque education system immigrant studentship is usually unevenly distributed between schools: in areas with a high immigrant population, immigrant students attend public schools, while most locals tend to enroll their children in privately funded schools which instruct their pupils almost entirely in Basque. Additionally, immigrant students in public schools tend to attend classes that are instructed mostly in Spanish, while locals enroll in classes predominantly instructed in Basque. This choice, which is ethnically guided, conflicts with one of the education system’s main objectives: to promote Basque language regardless of the ethnic, social, or linguistic origin of each student.

In Mirebe (please note that all names are pseudonyms), the town where the study took place, Spanish is the predominant language on the street while Basque is a minority language. In this paper we focus on Udabia, a secondary public school attended by a high proportion of immigrant students. More precisely, in the 2015/2016 school year the percentage of immigrant students at Udabia was 37%, while the average in Basque schools in that period was below 9%. In such an ethnically and linguistically diverse environment, teachers had to teach Basque to immigrant students, many of whom did not understand its importance within the Basque community. As illustrated in previously published results from this case study, some immigrant students bluntly refused to learn Basque, citing its lack of value to them, while their local counterparts did not show such resistance. In such cases, teachers often failed to manage the classroom effectively, as they were trying to teach Basque to an obviously unreceptive audience and the subsequent conflict seemed more significant to them than simple student misbehavior (Pérez-Izaguirre, 2018, 2019).

In this paper, student misbehavior is designated as transgression. Transgression in this context makes reference to the act of questioning the socially accepted rules of interaction, which is typical behavior in adolescent years. Apart from transgressions related to their refusal to learn Basque, some students in this school also transgressed other rules, by not complying with teachers’ instructions or respecting their peers. These transgressions involved students in relation to their peers and teachers within a specific power-relations framework. Such interactions also marked differences between the subjects involved, which had consequences for the constitution of their identities. In other words, identity was performed and represented in each of these transgressive interactions. Focusing on this idea, the aims of this paper are (i) to analyze the kinds of transgressions-as-interactions that can be classified in such a context, and (ii) to show how the concept of teenage transgression, as a type of interaction, constitutes a specific kind of identity. In line with the aims, the hypotheses I propose are (1) transgressions are negative for classroom environment, and (2) transgressions constitute the main problem when Basque learning is involved, as student identity is constituted in acrimonious interaction.

## Theoretical Framework

In this section, I will focus on two of the central elements of this paper: limits and transgression, the latter as a specific kind of interaction, and the concept of identity-in-interaction from a sociocultural perspective. The link between these two elements constitutes the main argument of this paper.

### Limits and Transgression

I will define limits, according to Bakhtin (1984), as the socially constituted rules or conventions embedded in everyday practices that mark and define what is expected from another person or group in a social situation. Based on this idea, transgression of limits leads to a clash of views with respect to what an individual or a group perceives is expected from them in a social situation. That is, when transgressions of these limits occur, the individual or group who perceives them feels they are inappropriate. Hence, limits are not necessarily related to ethnicity, gender or social class, but to the harmonious functioning of a social relationship.

A more recent contribution to transgression theory by Foley et al. (2012) specifies that transgressions should be contextualized in time and space, as what is considered in one place and epoch as transgressive might not be considered so in another. Transgression is located at the border of a norm bounded by limits, marked by what is appropriate. Thus, limits are the essential normative elements that separate appropriate from deviant behavior. In line with this idea, Jenks (2003, 2013) affirms that questioning or transgressing such limits means going beyond them, exceeding them and through such excesses, rules are reaffirmed. What is more, the internalizing of social order is based on such experience.

Many authors have claimed that in adolescence limit transgressions happen through different practices, such as the enactment of risky and violent behaviors (Bonino et al., 2005; Alarcón Bañares et al., 2010; León et al., 2010; Varela Garay et al., 2013; Krettenauer et al., 2014; Carrascosa et al., 2015; Cui et al., 2016; Patterson et al., 2016). In the education system, much research has been conducted on students’ discipline and disruptive behavior (Mooij, 2011; Wilson et al., 2011; Levin and Nolan, 2014; Garaigordobil and Martínez-Valderrey, 2016). A study on limit transgression was conducted by Hans (2008) in a French secondary education center where the discourse of a 15-year-old was analyzed. The study was conducted from a clinical psychoanalytical perspective and aimed to give further recommendations in the context of acrimonious teacher-student relationships. Hans (2008) claimed that in order to advance their academic and social abilities, subjects need to transgress or test what is already socially established. Through transgression, individuals develop their autonomy and creativity, as they must find harmony to internalize the new insight they have acquired.

In line with this idea, Eckert (2002, 2004) provides an insight into the so-called “teen-culture” of profanity and rebellious behavior in the American high school. According to her research, in adolescence, social order is internalized by testing social and institutional rules in the high school context, often through clothing, gesturing, and language profanity. In this process of differentiation many behavioral and psychological aspects interact, and these help adolescents reach a social position in society, which has consequences for their wellbeing (Kłym and Cieciuch, 2015). How such interactions constitute identity will be analyzed in the following section.

### Identity-in-Interaction

A sociocultural approach to the concept of identity-in-interaction by Bucholtz and Hall (2005) establishes that identity is produced through interaction and they define it is an emergent product of a linguistic game based on the “Self/Other” binary. In interaction, identities are intersubjectively constructed through complementary and overlapping relations, including similarity and difference. These identities are constantly shifting, through both deliberate and unconscious discourse, negotiated internally, and externally. Identities are also macro-socially, locally and temporarily embedded and subject to the status that individuals hold in society. This conceptualization of identity has a changing nature; it is internally and externally negotiated constantly.

In line with this idea, Jenkins (2008) suggests that identities are rooted in language, negotiated, flexible, and multi-dimensional. Identity shows the capacity of an individual to designate who is who and what is what, which implies a classification, evaluation, and hierarchy. Jenkins (2008) affirms that institutions provide specific channels for identity constitution, as contextual elements and social rituals are the basis for social relations. In these, power relations are present and institutions categorize individuals and groups, assigning each a specific role.

Dubet (2010) also acknowledges that identity is a consequence of experience. He explains that the unique experience of each individual builds his/her identity. In other words, the uniqueness of identity is provided by the capacity of each individual to accommodate such an experience into their self. Miles (2014) additionally argues that identity is dependent on the individual’s context and the power relations within these, as inequalities can occur between subjects involved in an interaction.

## Materials and Methods

This is a qualitative study using ethnographic methods. Ethnography is based on the long-term collection of discourses by and observation of people in the field (Erickson, 2010). My role as an ethnographer was to collect those discourses on voice-recorders or notebooks and transcribe them. I was positioned between students and teachers, which gave me an advantage in understanding each of the roles involved in the interaction, whilst maintaining a distance from them. This research followed the guidelines for personal data collection of the Ethics Committee for Research with Humans of the University of the Basque Country (Comité de Ética de Investigación con Seres Humanos de la Universidad del País Vasco, CEISH, UPV/EHU). All participants, including parents of minor students, signed an informed consent that enabled them to take part in this study.

### Data Collection

This investigation started in Spring 2015, when initial contact with Udabia was made. In July the Head of the School agreed to data collection beginning at the start of the next school year and preparatory documentary analysis began. For 2 months I collected data about the linguistic and social history of Mirebe, as well as the town’s immigrant population. In September 2015, participant observation started and lasted until June 2016. Participant observation is a technique designed to collect data about research participants in their natural environment and includes interaction with the ethnographer (Woods, 2012; Hammersley, 2018). At first, observation was only conducted with a class known at Udabia for their disruptiveness; this class also had the highest immigrant attendance in the school. Once observation had started, I realized that the most registered transgression was the act of questioning the rule, which was otherwise socially accepted, enforcing Basque learning: most immigrant students disliked the language or bluntly refused to learn it. Other transgressions were related to school rules and student-to-student disrespect, which caused conflict between them. In April 2016, observation was conducted in other classes with lower immigrant attendance and observation indicated that these students did not complain or refuse to learn Basque, but also disrespected each other and refused to comply with other rules of the school.

In spring 2016, interviews with students and teachers took place. These were designed to find out more about participants’ discourse regarding their own behaviors (Marvasti, 2010). I asked students why they acted transgressively in certain situations, and teachers how they felt and managed such transgressions. Finally, focus groups were proposed and conducted to analyze the discourse of students in a group (Morgan and Hoffman, 2010). Students were encouraged to interact with each other and answer cooperatively to issues that had been observed during the fieldwork, such as how they perceived their different kinds of transgressions.

### Analysis

Analysis took place after all the data was collected. Notes and voice-recordings were transcribed and saved as an RTF document, which was codified using Atlas.ti software, and families of codes were created. Following the aims of this paper, the classification of the kinds of transgressions as observed during fieldwork are (1) personal limit transgressions, (2) civic limit transgressions, and (3) social limit transgressions. Personal limit transgression makes reference to the act of questioning the socially accepted rules of interaction between peers, that is, disrespecting each other. Civic limit transgression involves non-compliance with school rules, such as disrespecting school services, challenging teachers’ authority, or asking teachers inappropriate questions. Finally, social limit transgression is a kind of civic limit transgression referring to community rule-breaking. Social limit transgression is different from the former, as it involves the non-compliance with a community rule that is naturalized for locals. In other words, a local would not usually transgress such a limit because it is naturalized for them, while a non-local could more easily transgress as they are not implicitly aware of its cultural importance.

### Research Participants

As mentioned, this is part of a larger study, but the sample utilized in this paper differs from previous publications (see Pérez-Izaguirre, 2018, 2019). In this case, the sample is composed of teachers (*N* = 4) and students (*N* = 6) engaged in interactions, which are analyzed from the students’ perspective, as they are the primary focus. These participants were selected because their interactions are relevant and explanatory for the main purpose of this paper: to analyze transgressions as interactions and their consequences for student identity formation.

The students selected were enrolled in the 2nd year of secondary education and were studying in a classroom composed of 19 students, of which 14 were immigrants. The relevant characteristics of the six students selected are represented in Table 1 according to their self-definition and age of arrival in the BAC.

**TABLE 1:** Students’ self identification and age of arrival in the BAC.

	**Self-identification**	**Age of arrival in the BAC**
Amaia	Latina, Ecuadorean	0
Agustín	Latino, Ecuadorean	1
Juan	Latino, Bolivian	7
Ana	Latina, Nicaraguan	12
David	Portuguese	12
Myriam	Bulgarian	12

*^1^In this paper I will only focus on international immigrants. Hence, students coming from different parts of Spain will not be considered.*

Four of the students self-defined as Latino, more precisely, Ecuadorean (Amaia and Agustín), Bolivian (Juan), and Nicaraguan (Ana). Two of these students had always attended a Basque school (Amaia and Agustín) and had had previous contact with Basque, whereas two of them had arrived in the BAC later, when they were 7 (Juan) and 12 (Ana). These four students’ mother language was Spanish. The other two students were European-descended, from Portugal (David) and Bulgaria (Myriam), and both had arrived in the Basque Country the previous year; Spanish was their second language.

## Results

### Personal Limit Transgressions

Personal limits as observed in this case study refer to boundaries between peers in relation to respect, interpersonal distance, and dignity. One of the most typically transgressed personal limits was interpersonal distance: in the initial classroom hours students were calm, but after recess they returned to class much more active. In this activeness, transgressions of personal space were usual: they tended to excessively touch and hit each other. In this paper I will designate these as physical interactions. Other transgressions involved insulting or mistreating each other. In this section, I will introduce three examples of such personal limit transgressions between peers.

#### Example 1

In one of the early hours of class, a teacher started to organize students to work in pairs. He asked two of the students who were close to him to work together:

Teacher 1: Amaia, pónte con David.

Amaia, work with David.

Amaia: ¿Qué?, ¿con esa chusma ingrata?

What? Do I have to work with this ungrateful riffraff?

The teacher probably did not hear Amaia’s comment, as he didn’t answer, and neither was there a reply from David, but he was obviously offended.

In this excerpt we can see how a teacher ordered Amaia to work with David. David was usually a silent and shy student and Amaia was considered one of the popular girls in this class. Amaia scornfully answered to the teacher’s command by insulting David, transgressing an obvious personal limit. This had an impact on David, who did not feel comfortable with the comment although it did not have any repercussions for Amaia.

#### Example 2

Insults also occurred when teachers were not present. During a focus group some Latin American students, Agustín included, told me how local Roma students called them racist names. A popular disrespectful term used in Spain for the last decade or so to refer to some Latin American people has been Machu Picchu, the name of the ancient Peruvian city. In the case Agustín described, local Roma students designated Ecuadorean pupils as “machupino,” a corruption of “Machu Picchu,” which is even more disrespectful. When local Roma students at Udabia called Ecuadorean student “machupinos” they intended, and succeeded, in causing offense. I was not present for such interactions, but Agustín expressed his frustration thus, “si supieran que eso es un monte…” (if only they knew that Machu Picchu is a mountain…). When racist insults were involved in an interaction, a personal limit was transgressed and the consequence was offense taken by the party being insulted.

#### Example 3

Juan was often particularly scornful and disrespectful to his peers, often refusing to talk to them. When they interacted with him, his response was often “Déjame en paz, tío” [Spanish] (*Leave me alone, dude*); or “Yo contigo no hablo” [Spanish] (*I don’t speak to you*). When questioned, he said his classmates behaved like children. Some of his peers felt very offended by Juan’s attitude toward them and decided not to interact with him anymore. In line with the previous two examples, Juan’s mistreatment of his peers transgressed personal limits.

Myriam, another student, showed great intuition interpreting attitudes such as the ones shown in examples 1, 2, and 3 and claimed that her classmates “…a veces… no se dan “de cuenta” de que… […] hacen daño (con sus insultos) [Spanish] (*don’t realize* […] *they hurt each other*) (*with their insulting comments*). In this sense, Myriam described accurately how students felt after such transgression between peers had taken place. However, it should be noted that on a few occasions these interactions were taken with humor and students were not offended by them.

### Civic Limit Transgressions

Civic limits, as registered in the field notes, allude to all the institutionally explicit rules regarding the smooth functioning of the school. I chose this term because it makes reference to the well-being and harmonious cohabitation of school community. Civic limits may comprise teachers’ roles as public servants, whose personal life should be kept separate, and their authority, as educators hold the legitimized power in the classroom. Teachers’ role includes guaranteeing a positive learning environment at school, and addressing inappropriate remarks toward them, or questioning their authority risks the smooth functioning of the school as an academic institution. When civic limit transgressions occurred, an appropriate classroom management method was key to keeping a positive classroom dynamic. In the following examples I will show three different examples of students transgressing civic limits.

#### Example 4

In this example, Teacher 2 was speaking about the food students ate in the canteen and Juan, who was used to pushing limits with both his peers and teachers, said “es que la comida del comedor, ¡qué asco!… quiero una pizza con su Coca-Colita” (*the food in the canteen is nasty!… I want pizza with a Coke*). After his interruption, Juan looked around to see if anyone had heard him. The teacher on this occasion ignored his comment but I, as an ethnographer, had heard and could not hide my surprise. When he realized I had heard he told me “¡Que era una broma!” (*That was a joke!*). Following Juan’s lead, Agustín added “Es que quiero comida americanita” (*I just want American food*). None of these comments were remarked on by Teacher 2.

In this case, the civic limit was present in the fact that school food was almost entirely a funded school service and such a right is guaranteed by public administration. When Juan and Agustín devalued the food at the canteen, they were expecting the teacher to react and engage in an acrimonious interaction. Although the latter did not happen, they deliberately transgressed a civic limit by showing disrespect for public goods (food), which was almost fully funded in an educational institution.

#### Example 5

This example took place in the first hour of class on a Tuesday. On Tuesdays, two teachers (Teacher 2 and Teacher 3) taught these students and they noticed that certain were missing, which was usual. Indeed, some students often missed the first hours of class because they claimed to be too tired. When the school bell rang and classes were supposed to change, the teachers and I remained to speak about the classroom dynamic for a while. Suddenly, Myriam, a student who had been missing for the first hour, arrived and after a look from Teacher 2 said: ¡Es que ya te lo he dicho! No me podía levantar (*I already told you! I couldn’t get up*). After this outburst, the two teachers and I remained silent, as we were shocked by her reaction. This teacher felt her authority being questioned by Myriam*’*s comment but did not react to it. It was obvious that Myriam had transgressed a civic limit by speaking in an inappropriate way to a teacher. As in Example 5, Myriam was probably expecting some reaction from the teacher but did not get any, at least directly.

#### Example 6

Teacher 2, who was involved in the previous two examples, instructed students to do some exercises.

Teacher 2: Si habéis hecho hasta el (ejercicio) 330, seguís hasta el final, si no (lo) habéis hecho, hacéis (también) hasta el final.

If you have completed exercise 330, keep going until the end, and if you have not finished it, you keep going until the end too.

Juan: ¿Me estás vacilando?

Are you kidding me?

After a brief and tense silence, Juan and the teacher ended up laughing. Later on, during the same lesson, Juan interacted with the teacher again:

Juan: ¿Tienes hijos?

Do you have children?

Teacher 2: Se me están quitando las ganas.

All of you are taking away the desire for it.

(Both Juan and the teacher laugh).

In this example two civic limit transgressions can be observed, both of them initiated by Juan. The first one takes place when Juan questioned her instruction, but the teacher did not take offense and laughed about it, letting the classroom dynamic flow. After a few minutes, Juan transgressed another civic limit by asking a personal question, transgressing an obvious boundary. By reacting with humor the teacher deflected conflict and maintained a positive classroom dynamic.

In examples 4, 5, and 6 good management of the classroom dynamic took place, as students transgressing a limit did not perceive that they provoked a negative reaction from teachers. Example 6 was especially remarkable, as the teacher humorously managed the limit transgression and the classroom environment remained positive.

### Social Limit Transgressions

Social limits are a kind of civic limits with particular characteristics. They are institutional rules that facilitate the harmonious functioning of school and as such, they are explicit norms. But at the same time, these norms are embedded and naturalized for most local students. As social limits are imbricated in community life, locals do not usually violate them, while for non-locals, these rules are not so obvious. Hence, under specific circumstances, non-locals transgress them. In this case study, these rules relate to Basque society and its education system, where both local and immigrant students were supposed to be integrated. One explicit rule is that Basque is the basic language in the public education system, and it is compulsory to study it. As such, it is also an implicit rule according to which students are classified and distributed, as classes are organized in terms of the level of Basque instruction. However, it is important to note that students (and their parents) choose the level of Basque they are instructed in. The six immigrant students who form the basis of these observations were enrolled in a classroom with a low Basque instruction and transgressed this social limit when they opposed Basque language learning, while local students involved in the larger ethnographic study did not. The latter also chose a higher level of Basque instruction.

Commonly, interactions where teachers tried to teach students words in Basque elicited blunt responses, such as “I don’t like the Basque language,” or “Why do we have to learn Basque?” In the following examples I will show indirect ways students tried to avoid speaking Basque or questioned its validity.

#### Example 7

During a Basque lesson, Teacher 4 tried to motivate students in various ways. Basque was especially difficult to teach, as students did not understand its importance in Basque schools and the community. It is also a difficult language to learn as it does not have many common elements with other languages. In this example, the teacher started the session by asking students about their ethnic origins:

Teacher 4: Nongoa zara? [Basque]

Where are you from?

Many students answered in Spanish and most seemed to be happy about the classroom dynamic. However, when the teacher started an explanation about Basque grammar the tone of students’ interventions changed.

Ana: ¿Qué?, Profesora, no entiendo. [Spanish]

What? Miss, I don’t understand.

Myriam: Jo, ¿qué? (? [Spanish])

Really, what?

In these interventions no specific question was posed and they were aimed at interrupting the explanation. They also led to a small boycott of the classroom dynamic and constitute transgressions in the sense that they aimed to prevent the Basque lesson from advancing. Although this type of behavior was occasionally observed in other subjects, it was most common during Basque lessons.

#### Example 8

This example also took place in a Basque class after some students had bluntly expressed their dislike of Basque. Teacher 4 felt offended and decided to ask one by one whether students liked Basque or not.

Teacher 4: Y a ti, te gusta (el euskera)?

Do you like Basque?

Ana: A mi antes no me gustaba lo del euskera y eso, pero ahora bien.

Before I didn’t like Basque and all that, but now it’s okay.

David: Me da igual.

I don’t care about it.

María: Normal

Normal

After these interactions, the teacher felt disappointed to see a lack of student investment in Basque. In contrast to the previous example, students demonstrated passivity, which frustrated their teacher. The limit transgression was initiated by students claim to dislike Basque, continued when the teacher felt the questioning of its validity and reacted to it, culminating in the students’ negative response. This transgression and teacher reaction led to a negative classroom environment.

#### Example 9

This example involves a complex history of interactions between Teacher 2 and Juan. Juan had observed many interactions between Teacher 2 and other students where the teacher reacted defensively toward complaints about learning Basque. In this case, in principle, Basque learning was not involved, but the teacher was unhappy with the results these students had obtained in an exam. After a tense interaction between the teacher and students, Juan, again testing limits, suddenly asked:

Juan: ¿A ti te gusta tu lengua (euskera)?

*Do you like your language* (*Basque*)*?*

Teacher: A ver, Juan, no se trata de que te guste o no te guste. Es mi lengua.

Juan, it is not about liking it or not. It is my language.

In this case, Basque learning was not directly involved but had been a matter of tension on other occasions, which Juan exploited. After the teacher showed irritation at the students’ low marks, Juan decided to bring up Basque language, out of the blue, probably to provoke the teacher even more. His interaction was aimed at bringing up a matter of conflict in the classroom. This indirect way of questioning the validity of Basque transgressed a social limit.

## Discussion

This paper has analyzed interactions in a multi-ethnic school setting. More precisely, it focused on the kinds of adolescent interactions between six adolescent students and four teachers in a highly ethnically diverse Basque school setting. Data shows that there are three kinds of interactions performed as transgressions. Transgression in this paper has referred to the act of using language or physical interactions to transgress a limit, which separated deviant from appropriate behavior. According to Foley et al. (2012), limits are contextualized in specific settings and in this case I have defined three kinds: personal, civic, and social limits. The first is limited by the interpersonal boundaries and dignity of student relations, the second is defined by the rules in a school as an academic institution, and the third is a specific limit related to respecting and promoting a local minority language. Following Jenks (2013), transgressions of these limits reaffirmed the norm and tension was produced in each of the examples. This tension sometimes resulted in a negative environment, whereas other occasions were answered with humor, promoting a positive classroom environment. Hence, the first hypothesis is refuted, as classroom environment was dependent both on the transgressions and the response to these: if the response was humorous, the classroom environment was prone to being positive.

Adolescent limit transgression, as observed in this case study, showed a need to mark difference and elicited a self-determined response from individuals in each of the examples presented. Following the concept of identity-in-interaction by Bucholtz and Hall (2005), identity was performed via transgression in each of these cases. Such excess was a performance of an identity that opposed the limit and I argue that it was a necessary experience for students. Experience was the basic element for adolescent identity constitution, as each experience was unique and each interaction differed, leading to a specific kind of identity transformation. In this case, identity was constituted in opposition to rules, using transgression as a tool to experiment with limits. When personal limits were transgressed, the socially accepted rules of interpersonal peer interaction were being actively resisted. Similarly, when civic limits were transgressed, institutional rules were directly or indirectly opposed, as explicit rules designed to facilitate the smooth functioning of the school were not respected. Opposition was also considerable in the case of learning Basque, as immigrant students transgressed this social limit of the school and teachers: to promote and protect Basque. Oppositions to each of the personal, civic, and social limits were necessary experiences in the identity formation of each of these subjects. Identity in each of these cases was performed in transgression, via opposition to the socially accepted rules of interaction, in each of their forms.

This paper has contributed to the theory of identity-in-interaction by introducing the concept of transgression to a Basque case study. More precisely, by analyzing transgressions of personal, civic, and social limits by six immigrant students in a Basque school, it concludes that transgressions constitute identities-in-opposition to the main rules of social interaction. Social limit transgressions were the most salient interactions in performing an identity, by resisting one of the main rules in Basque schools: learning Basque. These students represent common attitudes among immigrant students in this and other schools in the BAC more broadly, in contrast to local students who do not tend to transgress the rule of learning Basque. In fact, social limits for local students were naturalized and often unnoticed, making them unlikely to be transgressed. Social limit transgressions were noteworthy in this case study, as immigrant students refused or complained about learning Basque, the minority language that acts as an identity marker for a large part of the Basque community. This sometimes led to intercultural conflict, as teachers felt their work as promoters of Basque identity was undervalued or misunderstood. Hence, the second hypothesis is confirmed, as immigrant student identity was performed in transgression, which was an acrimonious intercultural interaction, and this constituted an obvious problem when Basque learning was involved.

A limitation of this study is that it does not analyze the social class disparities between immigrant and local students. Such a comparison could comprise the analysis of ethnic inequalities in relation to limit transgressions in academic contexts. Finally, this study contributes to the literature of second and third language acquisition in the case of immigrant students and the management of intercultural conflict in educational settings. Based on the results, I suggest that how teachers express sensitive personal responses to student transgressions related to the learning of Basque can improve classroom management and student learning. Finally, this study opens new avenues for research in multi-ethnic and multilingual environments when minority language learning is involved.

## Data Availability

The datasets for this study will not be made publicly available because according to the Ethics Committee that approved this research, data must remain confidential in all cases. This means that the author cannot share data with anyone (including any journal).

## Ethics Statement

Ethics Committee that approved the study: Comisión Ética de Investigación con Seres Humanos de la Universidad del País Vasco (CEISH, UPV/EHU). Ethics Committee for Research with Humans of the University of the Basque Country. Consent procedure: Research participants were informed of the research project. The school agreed to data collection and participants were given and signed an informed consent in order to take part in this study. Please note that confidentiality of participants is kept throughout all the article and these remain anonymous (pseudonyms are used).

## Author Contributions

The author confirms being the sole contributor of this work and has approved it for publication.

## Conflict of Interest Statement

The author declares that the research was conducted in the absence of any commercial or financial relationships that could be construed as a potential conflict of interest.
